# Parasitic contamination in green vegetables from open markets in Gampaha District, Sri Lanka: Implications for human health

**DOI:** 10.1371/journal.pone.0321853

**Published:** 2025-04-29

**Authors:** Nayana Gunathilaka, Rashmi Kavindya, Kithsiri Gunawardena, Deepika Amerasinghe

**Affiliations:** 1 Department of Parasitology, Faculty of Medicine, University of Kelaniya, Ragama, Sri Lanka; 2 Department of Zoology and Environmental Management, Faculty of Science, University of Kelaniya, Dalugama, Sri Lanka; Niigata University of Pharmacy and Medical and Life Sciences, JAPAN

## Abstract

Intestinal parasitic infections in Sri Lanka have received little attention due to their perceived low prevalence. Consuming raw vegetables without proper washing is one of the main ways to transmit intestinal parasitic infections. Therefore, this study investigated the contamination of parasites in vegetables. A cross-sectional study was conducted from August 2023 to January 2024 at fifty selected open markets in ten cities (Ragama, Miriswatta, Kirillawala, Kadawatha, Balummahara, Kiribathgoda, Peliyagoda, Weliweriya, Imbulgoda and Gampaha) in Gampaha District, Sri Lanka. Four vegetable types, namely *Centella asiatica* (Gatu kola) [n = 162], *Ipomoea aquatic*a (Kankun) [n = 150], *Alternanthera sessilis* (Mukunuwenna) [n = 160] and *Lactuca sativa* (Lettuce) [n = 148] that are consumed either raw or half cooked were selected. Approximately 50 g of each vegetable was taken randomly at each sampling attempt. Each sample was dipped in the Tween 20 (5%) in a shaker for 15 minutes, followed by sedimentation overnight and centrifugation (2000 × g for 15 min). Both supernatant and sediment were examined under a light microscope to detect parasitic stages. The overall prevalence of parasitic contamination in the samples was 21.29% (n = 132). *Centella asiatica* indicated the highest contamination (37.65% [61/162]). *Lactuca sativa* had the lowest contamination (2.02% [3/148]). Parasites including protozoan (*Entamoeba histolytica, Giardia lamblia, Toxoplasma gondii, Blastocystis hominis*, *Paramphistomum* spp and *Balantidium coli*) and helminths (*Ascaris* spp*., Hymenolepis* spp*., Strongyloides stercoralis, Trichuris* spp, *Taenia* spp, *Toxocara canis* and hookworms) were detected. *Blastocystis hominis* was the most predominant (4.68%; n = 29). *Toxocara* sp, *G. lamblia* and *Eimeria* sp were identified as least abundant (0.77%; n = 5). Every sample contained at least one parasitic contamination. A significant difference between the type of vegetables and the existence of parasites was identified (*P* = 0.008). The parasitic stages identified could cause infections among people with poor food hygienic/ preparation practices. Therefore, health authorities must educate consumers on precautionary measures to prevent re-emerging intestinal parasitic infections in Sri Lanka.

## Introduction

Intestinal parasites are among the key public health issues, especially in tropical and subtropical countries [1]. Consumption of raw or undercooked vegetables plays a vital role in transmitting food-borne parasitic diseases to humans [2]. The recovery of parasites from fresh-sold vegetables and fruits could indicate the quality of the cultivation process, irrigation, and post-harvest handling [3].

Despite ten years of mass deworming in Sri Lanka between 1994–2005, an exceptionally high prevalence of intestinal nematode infection was identified among plantation sector communities [4]. The case incidence has dramatically decreased over the last two decades due to control efforts and improvements in the living standards of communities [5]. However, some case reports show that infections are still common in some regions of the country with low economic status [6]. Therefore, intestinal parasitic cases in Sri Lanka cannot be ignored until the diseases are eliminated.

Common intestinal parasites found in Sri Lanka include protozoa such as *Giardia intestinalis* and *Entamoeba histolytica*, as well as helminths like roundworm, *Ascaris lumbricoides*, whipworm, *Trichuris trichiura*, pinworm, *Enterobius vermicularis* and species of hookworms [6]. These parasites are transmitted through contaminated food, water, or soil, poor personal hygiene, and improper waste disposal. Contaminated fresh fruits and vegetables will become an entry mode for intestinal parasites due to improper handling and storage, not maintaining food hygiene when preparing, inappropriate washing or consuming them raw or undercooked [7]. Fresh vegetables are a common dietary component alongside rice, which is the staple food of Sri Lankans. The World Health Organization recommends the consumption of vegetables and fruits for the benefits of a balanced diet that protects people from chronic diseases, micronutrients, and supplement gain, which is ultimately helpful in maintaining a healthy lifestyle [8–10]. However, consuming unwashed raw vegetables and fruits can significantly contribute to food-borne illnesses. Many of the fresh vegetables available in markets are cultivated in community gardens, making them susceptible to contamination at various stages, including production, distribution, transportation, storage, and ultimately reaching the consumer [11]. It is not only inadequate hygiene practices from the producers to the point of sale but also the use of animal manure to enrich agricultural soil and the irrigation process with wastewater that can significantly contribute to food contamination, particularly in the case of horticultural products [9,11]. Furthermore, the infective stages of many intestinal parasites are commonly found in the soil and the environment, making it easy for them to transfer to vegetables during the cultivation process.

Several studies conducted worldwide have identified that common contaminants in the vegetables in market samples were *A. lumbricoides*, *Cryptosporidium* spp., *E. histolytica*, *E. vermicularis*, *G. intestinalis*, species of hookworm, *Hymenolepis* spp., and *T. trichiura, Toxocara* sp., *Taenia* sp. and *Strongyloides stercoralis* [12–19]. Contamination of these vegetables with parasites may be attributed to the water used to moisten vegetables and post-harvest handling [14,15,20]. As a result of the low prevalence rate of intestinal parasitic infections in the country, the perception of the general public has become low. Therefore, this may become a limitation in eliminating intestinal parasitic diseases from the country. No published study is available on the presence of intestinal parasitic stages in green vegetables sold in open markets, and a survey of this nature would be beneficial to estimate exposure risk to intestinal parasitic infections in the Sri Lankan community. Hence, the present study aimed to understand the presence of intestinal parasites in fresh horticultural products sold at an open central market in the Western province of Sri Lanka.

## Materials and methods

### Study design and setting

**Fig 1:**
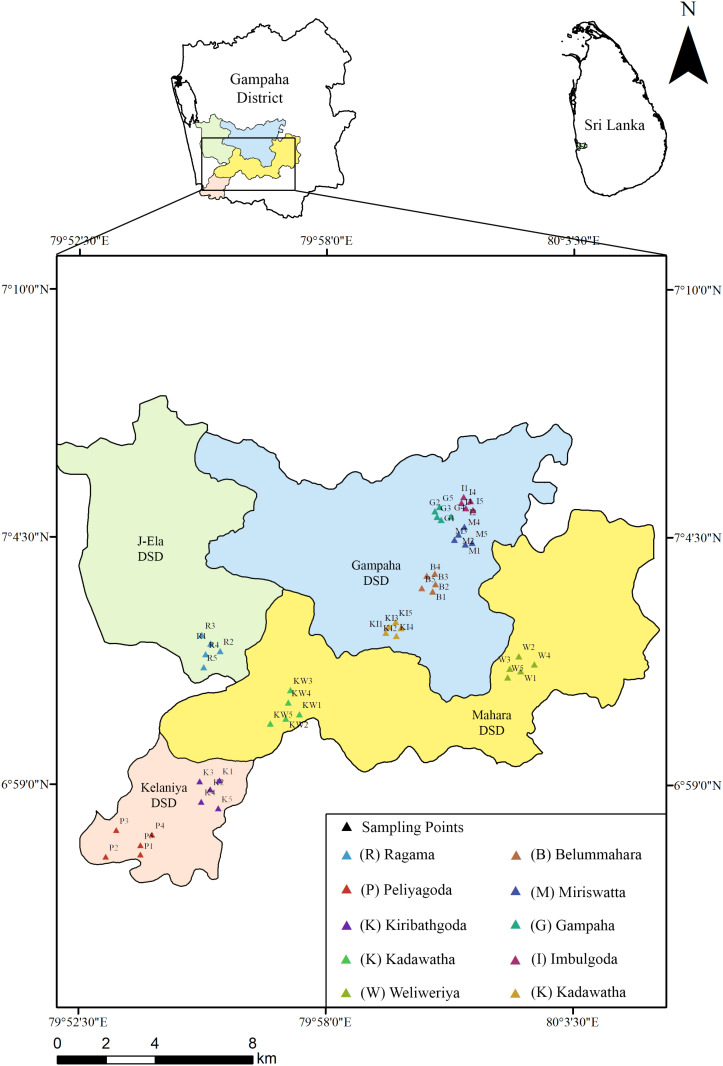
The map showing the open markets selected for sampling in the present study.

A prospective cross-sectional study was conducted in Gampaha District from August 2023 to January 2024. A total of 50 central open markets as five markets per city, which collect vegetables and fruits from different parts of the country and re-distribute them to other places that are located in ten main cities (Ragama, Miriswatta, Kirillawala, Kadawatha, Balummahara, Kiribathgoda, Peliyagoda, Weliweriya, Imbulgoda and Gampaha) in Gampaha District, Western Province of Sri Lanka were selected for this study (Fig 1). Ethical clearance for this study was not relevant since this investigation did not involve humans or animals as study participants.

### Collection of samples

**Fig 2:**
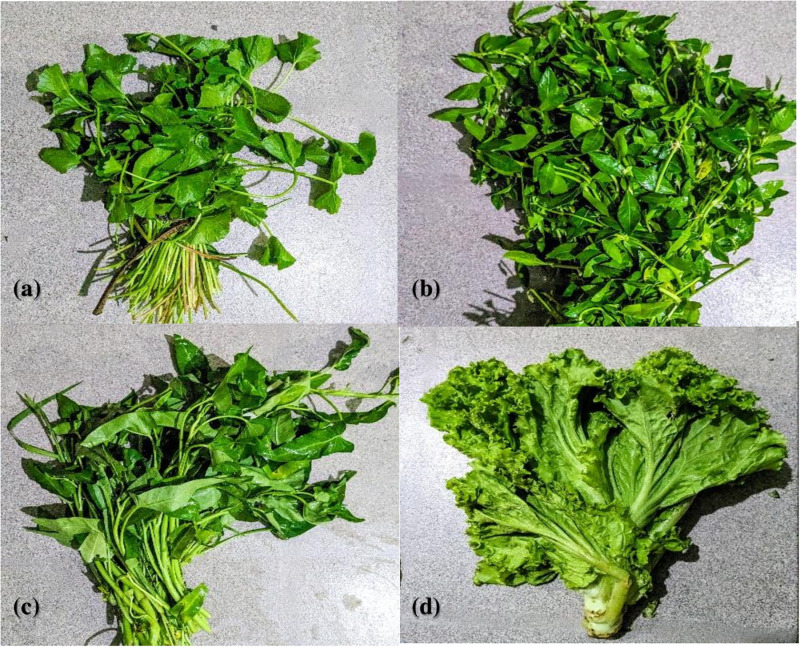
Four different types of tested fresh green leafy vegetables. (a) *Centella asiatica* (Common name: Gotu kola). (b) *Alternanthera sessilis* (Common name: Mukunuwenna). (c) *Ipomoea aquatic*a (Common name: Kankun). (d) *Lactuca sativa* (Common name: Lettuce).

Five different types (**Indian pennywort** (Gotu kola) [*Centella asiatica*], **sessile joyweed** (Mukunuwenna) [*Alternanthera sessilis*], **Chinese morning glory** (Kankung) [*Ipomoea aquatic*a], and **lettuce** [*Lactuca sativa*]) of frequently consumed green leaves that are consumed either raw, steamed or half cooked were selected for sampling (Fig 2). Approximately 50 g of each item chosen was randomly collected from sellers at the above-selected open market at each sampling attempt. The fresh vegetable samples were collected in clean, labelled plastic bags and transported to the Department of Parasitology, Faculty of Medicine, University of Kelaniya, Ragama, Sri Lanka, for parasitic examination.

### Detection of parasitic stages in the open market samples

Fresh vegetable samples (50 g) were washed using 150 mL of 5% Tween 20 solution for detaching the parasitic stages, eggs and larvae of helminths and cysts, and oocysts of protozoan parasites that are presumptive to be associated with vegetable contamination [12]. The vegetables dipped in the tween 20 solution were kept in a shaker for 15 minutes. The washing was kept for 24 hours for sedimentation and centrifuged at 2000 × g for 15 min. The sediment and supernatant were examined separately under a light microscope to detect parasitic stages.

### Data analysis

All the data were entered into a Microsoft Excel worksheet, and all the data analysis was performed using the IBM SPSS software package (Version 24.0). The data were analyzed using Chi-square tests to determine the association between different vegetable types and the prevalence of various parasitic species. The significant p-value threshold was set at 0.05. The species abundance data in other locations were analyzed using One-way ANOVA at a 95% confidence level, followed by Tukey’s pairwise comparison in SPSS (version 24.0). A p-value < 0.05 was considered statistically significant. In addition, the Bray-Curtis similarity-based cluster analysis was utilized to identify the overall clustering status of parasites from different areas regarding the prevalence of different parasitic species. Further, Distance-Based Redundancy Analysis (dbRDA) was also performed to highlight and visually represent the underlying segregation patterns of sites due to the occurrence of different parasites in the collection sites using Plymouth Routines in Multivariate Ecological Research Variation 6 (PRIMER 6).

## Results

### Prevalence of parasitic contamination in vegetable samples

From the 620 fresh vegetable samples examined, 21.3% (n = 132) were positive for at least one parasitic contamination. *Centella asiatica* showed the highest contamination rate (37.65% [61/162]), while *L. sativa* had the lowest (2.02%) contamination. Several helminths and protozoan parasite species were isolated from different vegetable types collected at various locations. Eggs of hookworm species, *Blastocyctis hominis*, trematode and *Ascaris* species were encountered as predominant organisms (Table 1).

**Table 1:** Relative abundance of intestinal parasites contaminated in vegetable types tested.

Parasitic species	Leaf type								Percentage of positive samples (%)
	*Centella asiatica*		*Alternanthera sessilis*		*Ipomoea aquatica*		*Lactuca sativa*		
	No. positive	Relative abundance	No. positive	Relative abundance	No. positive	Relative abundance	No. positive	Relative abundance	
Hookworm	11	18.03	6	13.95	4	16	0	0	16.15
*Ascaris* sp.	6	9.84	3	6.98	1	4	0	0	7.69
*Balantidium coli*	0	0	2	4.65	1	4	0	0	2.31
*Entamoeba* sp.	1	1.64	1	2.32	0	0	0	0	1.54
*Giardia lamblia*	0	0	0	0	1	4	0	0	0.77
*Hymenolepis* sp.	1	1.64	1	2.32	0	0	0	0	1.54
*Paramphistomum* sp.	0	0	3	6.98	2	8	1	33.34	4.62
*Toxoplasma gondii*	1	1.64	2	4.65	0	0	0	0	2.31
*Strongyloides stercoralis*	5	8.19	3	6.98	2	8	0	0	7.69
*Toxocara* sp.	0	0	0	0	1	4	0	0	0.77
*Trichuris* sp.	2	3.28	1	2.32	2	8	0	0	3.85
*Taenia* sp.	2	3.28	1	2.32	0	0	0	0	2.31
*Blastocystis hominis*	14	22.95	8	18.60	5	20	2	66.67	22.31
Trematode egg	10	16.39	11	25.58	5	20	0	0	20.00
*Eimeria* sp.	0	0	0	0	1	4	0	0	0.77
*Trichostrongylus* sp.	7	11.48	0	0	0	0	0	0	5.38

Protozoan parasites included *E. histolytica* (0.32%; n = 2), *G. lamblia* (0.16%; n = 1), *Toxoplasma gondii* (0.48%; n = 3), *B. hominis* (4.68%; n = 29), *Paramphistomum* spp (0.81%; n = 5), and *Balantidium coli* (0.48%; n = 3). Helminthic parasites, namely *Ascaris* spp. (1.61%; n = 10), *Hymenolepis* spp. (0.32%; n = 2), *S. stercoralis* (1.61%; n = 9), *Trichuris* spp. (0.81%; n = 5), *Taenia* spp. (0.48%; n = 3), *Toxocara canis* (0.16%; n = 1) and hookworms (3.39%; n = 2) were detected. *Blastocystis hominis* was the most predominant parasite found in 4.68% of the samples (n = 29). *Giardia lamblia* and *T. canis* were the parasites that were detected the least in this study. Trematode eggs were observed predominantly from *A. sessilis* and *I. aquatic.* Importantly, every sample tested contained at least one type of parasitic contamination, highlighting the widespread intestinal parasites in the tested vegetables (Table 1).

**Fig 3:**
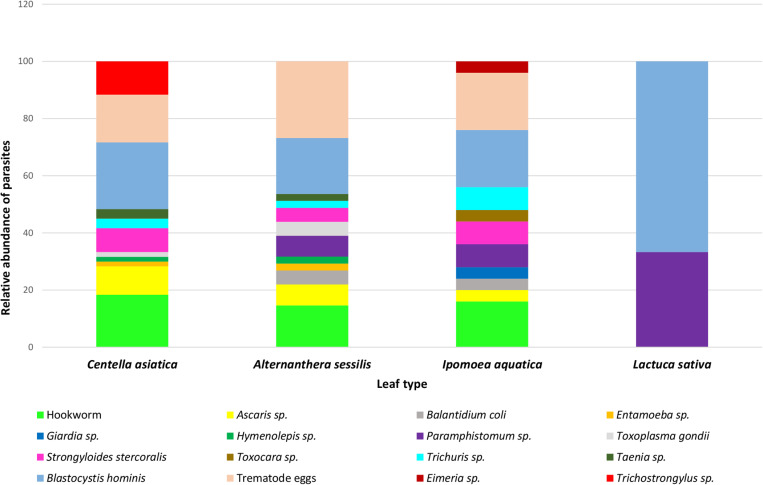
Relative abundance of parasites encountered from different types of green vegetables in this study.

A graphical illustration of the relative abundance of parasitic species encountered from different vegetables tested in the present study is illustrated in Fig 3. The chi-square analysis confirmed a significant association between vegetable types and the prevalence of certain parasites (*P* < 0.05). This suggests that the type of vegetable can influence the likelihood of parasitic contamination, which could be attributed to factors such as different cultivation, handling, and environmental exposure.

### Variations of parasite species encountered from different locations

**Fig 4:**
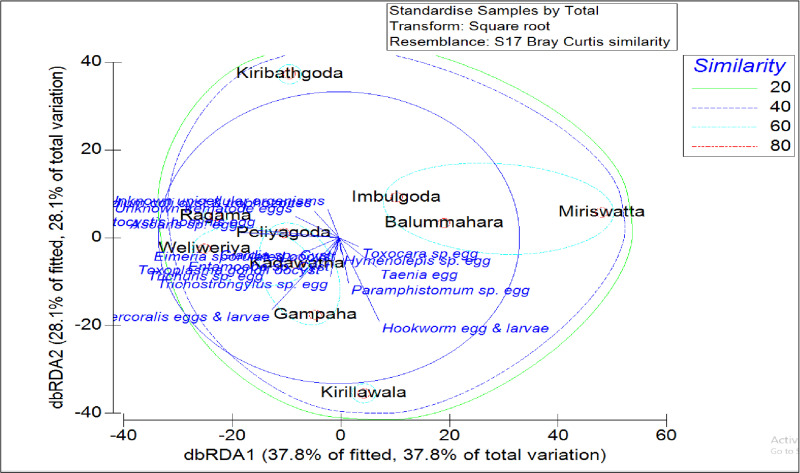
dbRDA plot depicting the spatial variation of parasitic contamination in leafy vegetables with different sampling locations. *Note:* The length of the arrows represents the explanation power of the characteristics for the features in the matrix. The relative position of the samples to the direction of the axis describes the relationship of the sample with the characteristic.

The clustering pattern of the parasitic species encountered from ten different locations is indicated in Fig 4. According to the dbRDA plot, the overall parasitic prevalence in the market samples collected from Ragama, Peliyagoda, Weliweriya, Kadawatha and Gampaha clustered together. Kiribathgoda is shown as an isolated cluster. Imbulgoda, Balummahara, and Miriswatta locations clustered together with 60% similarity (Fig 4).

**Fig 5:**
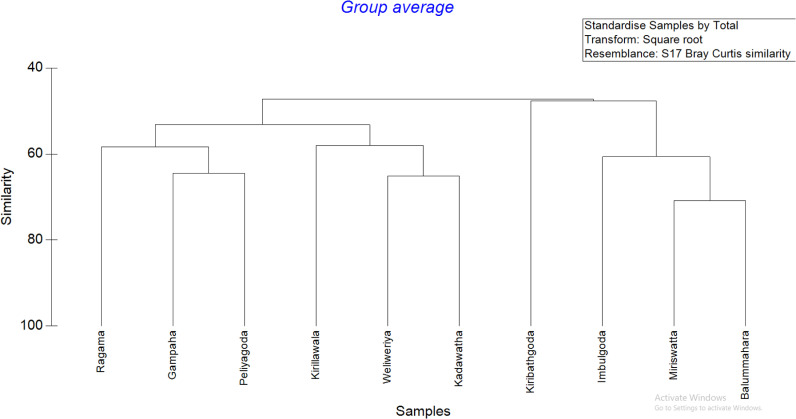
Dendrogram showing the spatial clustering of study sites based on the prevalence of parasites in leafy vegetables.

According to the Bray-Curtis similarity clustering, three main clusters: ***cluster 1***- (Ragama, Gampaha, Peliyagoda, Kirillawala, Weliweriya and Kadawatha), ***cluster 2***- Kiribathgoda, ***Cluster 3***- (Imbulgoda, Miriswatta, Balummahara) were denoted (Fig 5). According to the ANOVA and Tukey’s pairwise comparison, it was confirmed that the presence of parasitic stages in the leafy vegetables considered in this study was significantly different (*P* < 0.05) from the sampling locations, especially in the three clusters identified in this study.

## Discussion

The findings indicate a notable contamination rate of 21.29% among the vegetable samples tested, with *C. asiatica* exhibiting the highest contamination rate and *L. sativa* showing the lowest. This contamination might arise from the leaves’ capacity to trap parasites within their layers and uneven surfaces, which offer convenient sites for parasite adherence. Also, the distance between the edible part of the leaf and the soil is smaller than other leaf types. Generally, in most markets, *C. asiatica* is usually sold with roots and stalks, and that could increase contamination via soil. However, many studies indicated that *L. sativa* has the highest parasitic contamination [15,16,21]. The low contamination indicated in the present study could be because most *L. sativa* cultivations are cultivated commercially through hydroponics cultures with special care, thereby reducing the chance of receiving parasites with soil contact.

The presence of medically important parasites, encompassing protozoan and helminthic organisms, suggests a diverse range of potential health hazards associated with vegetable consumption. Previous studies in other developing countries, including Iran, Iraq, Nigeria, Pakistan, and Egypt, indicated higher parasitic contamination than in the present study [22–23]. However, studies conducted in developing regions such as Saudi Arabia [24] and Turkey [25] indicated lower parasitic contamination. Differences between percentages may be due to differences in the living standards of the communities, cultivation methods, climatic changes, and pre-post-harvesting care.

The predominance of *B. hominis* among the detected parasites is consistent with previous research highlighting its prevalence in Brazil [26], Mexico [27], Iran [28], Iraq [29] and Egypt [30]. However, a similar study conducted in the East of the Nile Delta in Egypt indicated that *B. hominis* was the least common parasite among the vegetable samples tested [31]. This protozoan organism, known to cause gastrointestinal symptoms in infected individuals, represents a significant public health concern due to its widespread presence in the tested samples. The possible contamination of this parasite with the vegetables could be related to the irrigation of contaminated water or environmental contamination by animal feces [30].

In the present study, some unidentifiable trematode eggs were also detected from the vegetable samples collected, notably from *C. asiatica* and *A. sessilis*. The highest positive samples were detected with trematode eggs (20%). Some studies conducted in Bangladesh and Malaysia have also indicated the presence of unidentified trematodes form market samples of *Persicaria odorata* (daun kesum) [32,33]. The presence of trematode contamination could be characterized by high temperatures since the temperature substantially impacts the emergence, survival, and infectivity of trematodes [34]. Therefore, the increases in environmental temperatures could lead to a distinct rise in trematode emergence [35].

In this study, hookworms were detected [16.15% (21/130)] as the second predominant parasite in the vegetables examined. These findings were in agreement with previous studies published in Thailand [2,36], Ghana [13], Sudan [15] and Iran [17]. However, a study conducted in Egypt has indicated a low prevalence (1.1%) [30] and several studies with no hookworm egg contaminations in market samples [12,14,37–39]. Differences in the prevalence of hookworms may be due to changes in the geographical location, climate conditions, and the types of soil [2,40]. However, the high contamination of hookworm eggs in vegetables in the present study may be associated with poor sanitation or using contaminated water for irrigation [2]. Therefore, lack of proper footwear and skin exposure to contaminated soil would be responsible for hookworm infection in humans.

In this study, eggs of *Ascaris* species were noted as one of the leading parasites in the examined vegetables. *Ascaris* eggs are among the most common parasites contaminating vegetables and other foods [41]. They are a priority for management due to their widespread impact on global populations via oral transmission [42]. These eggs are often found in environmental samples contaminated with fecal matter, such as water; however, they quickly settle out of the water column and reside in the soil, where they can be transmitted to growing plants and individuals who come into contact with infected soil. Consequently, human infection rates likely influence the high incidence of *Ascaris* eggs in plants and soil. This high prevalence may be attributed to the remarkable fecundity of adult female *Ascaris*, which lays approximately 200,000 eggs daily, maximizing its chances of transmission to subsequent hosts. Moreover, the thick-shelled nature of *Ascaris* eggs renders them more resistant to various adverse conditions, such as chemical exposure and dehydration, allowing them to withstand harsh environmental conditions and persist longer in soil habitats necessary for their growth [43].

Additionally, protozoan parasites such as *E. histolytica* and *G. lamblia*, along with various helminthic parasites including *S. stercoralis*, further underscore the complexity of parasitic contamination in green vegetables. *Giardia lamblia* contamination was detected only from *I. aquatica,* which has a prevalence of about 0.77% among the positive samples. Some studies conducted in other countries have reported the prevalence of this species between 6–35% among green vegetables [38,44]. This prevalence could be attributed to the ability of *G. lamblia* to survive under cool and moist conditions in cyst form and its resistance to chlorinated drinking water. *Balantidium coli* was identified from *I. aquatica* and *A. sessilis*. This has been the most prevalent species of parasite in some studies conducted in Cameroon [45] and Ghana [46]. The contamination in the present study may be due to irrigated water contaminated with human or pig faecal matter [46].

This study identified *Toxoplasma gondii* as the second most prevalent protozoan parasite. Similar studies conducted in Gaza, Palestine [47], Marrakech, Morocco [48], and Canada [49] have also indicated that *T. gondii* contamination in leafy vegetables. The primary source of contamination of this parasite could be the contamination of the environment by infested stray cat feces [47]. *Strongyloides stercoralis* was also detected in this study. This species has a complex life cycle with free-living stages in the environment that do not require a host for its proliferation [50]. The prevalence of this parasite was noted as 7.69% of the positive samples, which was observed as low compared to the previous studies conducted in Ghana (43%), Ethiopia (21.9%) and Thailand (10.6%) [2,13,51]. In addition, this study indicated the contamination of vegetables with *Toxocara* spp eggs. This species may cause human toxocariasis, a zoonotic infection caused by larval stages of *T. canis* and, less frequently, by *T. cati* [52]. However, the present study indicated a low prevalence of the parasite compared to previous studies done in Iran [53], Libya [38], southern Ethiopia [14], Vietnam [12], and Turkey [37]. As reported by several studies, domestic animals could be the source of contamination for *Toxocara* eggs. Long-term survival outside their hosts and high fecundity may be responsible for soil contamination with infective eggs [2,52].

In Sri Lanka, cow dung and other animal wastes are widely used to cultivate leafy vegetables. Some previous studies published in Sri Lanka as well as in other countries have highlighted the parasitic contaminations in dung, such as hookworms (*Bunostomum* spp.), whipworms (*Trichuris* spp.), amphistomes, cestodes (*Moniezia* spp.), Trematodes and oocysts of coccidia [54–58]. Therefore, the possibility of contamination in tested vegetables with *Paramphistomum* sp., *T. gondii*, trematode eggs and *Trichuris* sp. could be due to the parasitic contamination associated with the animal faeces used in cultivation as organic fertilizer or other possible contaminations at the cultivation sites.

*Trichostrongylus* species, abundant among herbivores, including cattle and sheep, have indicated a possibility of human infections [59], especially by contaminated vegetables/water and poor sanitary conditions in rural areas [60]. Therefore, the presence of *Trichostrongylus* sp. among tested samples could indicate the possibility of risk of human infection among consumers. In agreement with the present study, previous studies have also detected *Hymenolepis* sp, namely, *H. nana* eggs in watercress samples, while *H. diminuta* eggs in lettuce samples [16,24,44,61]. The prevalence rate of the above parasites ranged from 2.4–14.5% in previous studies. However, the present study indicated that *Hymenolepis* sp eggs were only from *A. sessilis* and *C. asiatica,* with a prevalence rate of 1.54% of the total samples positive for parasitic contamination.

*Taenia* spp. are important tapeworm species in humans and domesticated animals, possibly leading to a substantial health and economic burden [62]. Humans are the sole definitive hosts of three zoonotic *Taenia* spp., namely, *T. solium*, *T. saginata* and *T. asiatica* [63]. The prevalence of *Taenia* spp. eggs found in fruits and vegetables is high, ranging from 0.9 to 43% [17,38], posing a consumer risk [62]. The present study also indicated the presence of *Taenia* sp. eggs in *C. asiatica* and *A. sessilis*. Therefore, this could also be due to the contamination of animal wastes at the cultivation sites or the storage facility.

Bray-Curtis similarity clustering results revealed distinct groups of locations with similar patterns of parasitic contamination, highlighting spatial variations in parasitic prevalence among different sampling sites. The differences between markets may be because of varying vegetable sources or hygienic practices in handling and washing by different sellers [2]. The results in the present study highlight that raw vegetables from the markets in the study areas can act as possible vehicles for parasitic transmission to humans. These findings also highlight the importance of considering spatial dynamics and local context in assessing and addressing parasitic contamination risks associated with leafy vegetable consumption.

Furthermore, to reiterate the appropriate washing process, a study conducted in Iran has denoted that the pre-washing procedure with tap water or underground water may not eliminate parasites from vegetables [53]. Therefore, health authorities should provide knowledge of proper washing methods for local people to prevent parasitic transmission. Hence, policymakers and public health authorities can develop targeted interventions to mitigate the spread of parasitic infections and ensure the safety of leafy vegetable consumption across different locations.

Despite improvements in public health and food safety measures, the risk of intestinal parasite transmission through contaminated fresh vegetables remains a significant concern, particularly in regions with inadequate hygiene and sanitation practices. Gaps in legislation and policy enforcement contribute to the persistence of this issue, highlighting the need for stricter regulatory oversight, standardized testing protocols, and better integration of food safety measures with public health initiatives. Best practices in production and post-harvest handling, such as using uncontaminated irrigation water, maintaining hygienic harvesting techniques, and improving storage conditions, are essential to reducing parasite contamination at the source. Additionally, consumer practices are crucial in mitigating risks, with proper washing, safe cooking methods, and awareness campaigns being key to minimizing exposure. Research opportunities exist to explore epidemiological trends, assess decontamination interventions, and develop molecular detection techniques for improved parasite surveillance. Addressing these challenges through policy reforms, improved agricultural practices, consumer education, and scientific advancements is critical to ensuring food safety and reducing the burden of intestinal parasites.

It is essential to acknowledge the limitations of this study. The sampling bias, the limited scope of parasite detection methods, and other factors may influence the generalizability of the findings. The occurrence of parasites and soil-transmitting helminth contamination rate is associated with climate and temperature [2]. Generally, higher parasitic contamination rates could be identified during the warm and cold seasons [17,23,64]. The present study did not consider seasonality, which could be emphasized as one of the limitations of this study. Further, this study did not consider the intensity of vegetable washing before selling and the source of water used for washing. However, addressing these limitations in future research endeavors could enhance the comprehensiveness and robustness of findings related to parasitic contamination of vegetables, ultimately contributing to more effective strategies for food safety and public health protection.

## Conclusion

In conclusion, the findings of this study, supported by comparisons with published literature, emphasize the importance of enhancing food safety practices and promoting consumer awareness to mitigate the risk of parasitic infections associated with vegetable consumption. Addressing this issue requires multifaceted approaches encompassing improved hygiene standards, regulatory measures, and community education initiatives to ensure the safety and quality of vegetable products and protect public health.

## Abbreviations

WHO: World Health Organization
SPSS: Statistical Package for the Social Sciences
ANOVA: Analysis Of Variance
dbRDA: Distance- Based Redundancy Analysis
PRIMER: Plymouth Routines in Multivariate Ecological Research
STH: Soil – Transmitted Helminths
